# Hybrid Carbon Black/Silica Reinforcing System for High-Performance Green Tread Rubber

**DOI:** 10.3390/polym16192762

**Published:** 2024-09-30

**Authors:** Muhua Zou, Wenke Gao, Zengcai Li, Binghua Liu, Bingxiang Li, Kai Liu, Jinhui Liu

**Affiliations:** 1 Key Laboratory of Rubber-Plastics, Ministry of Education/Shandong Provincial Key Laboratory of Rubber-Plastics, Qingdao University of Science and Technology, Qingdao 266042, China; 2203070135@mails.qust.edu.cn (M.Z.); wenkegao0502@163.com (W.G.); 19819612709@163.com (Z.L.); liukai91@pku.edu.cn (K.L.); 2 Qingdao Dongyi Heating Management Co., Ltd., Qingdao 266100, China; 13884940444@163.com; 3 The Yellow River Delta Chambroad Institute Co., Ltd., Binzhou 256500, China; lbx901110@163.com

**Keywords:** hybrid reinforcing fillers, carbon black, silica, high-performance green tire, dispersion uniformity

## Abstract

Silica, as a high-quality reinforcing filler, can satisfy the requirements of high-performance green tread rubber with high wet-skid resistance, low rolling resistance, and low heat generation. However, the silica surface contains abundant silicon hydroxyl groups, resulting in a severe aggregation of silica particles in non-polar rubber matrix. Herein, we explored a carbon black (CB)/silica hybrid reinforcing strategy to prepare epoxidized natural rubber (ENR)-based vulcanizates. Benefiting from the reaction and interaction between the epoxy groups on ENR chains and the silicon hydroxyl groups on silica surfaces, the dispersion uniformity of silica in the ENR matrix was significantly enhanced. Meanwhile, the silica can facilitate the dispersity and reinforcing effect of CB particles in the ENR matrix. By optimizing the CB/silica blending ratios, we realized high-performance ENR vulcanizates with simultaneously improved mechanical strength, wear resistance, resilience, anti-aging, and damping properties, as well as reduced heat generation and rolling resistance. For example, compared with ENR vulcanizates with only CB fillers, those with CB/silica hybrid fillers showed ~10% increase in tensile strength, ~20% increase in elongation at break, and ~20% increase in tensile retention rate. These results indicated that the ENR compounds reinforced with CB/silica hybrid fillers are a promising candidate for high-performance green tread rubber materials.

## 1. Introduction

The rapid development of the automobile and transportation industry has triggered a series of severe problems, such as environmental pollution caused by waste tires and particles and resource depletion caused by increased fuel consumption [1,2]. Traditional tread rubber materials are typically reinforced using carbon black (CB) [3,4,5]. CB is mainly extracted from petroleum hydrocarbons or coal tar. For every ton of carbon black produced, approximately 1.8~2.3 tons of crude oil are needed [6,7]. Meanwhile, during the driving process of a vehicle, the tires are subjected to deformations due to the pressure, which causes severe friction between rubber and fillers as well as among fillers [8]. This leads to a sharp increase in energy consumption and heat generation of rubber materials, producing various oxides such as CO_2_, NO, NO_2_, and SO_2_, which results in serious environmental pollution [9,10]. Since the implementation of the EU tire label, the demand for green tires with high humidity and slippery, low-rolling resistance, and low-raw heat is increasingly urgent [11,12]. White carbon black, also known as silica, is an important high-quality reinforcing filler, and its composition is represented by SiO_2_·nH_2_O [13]. The silica is prepared from quartz sand and in a non-carbon process [14]. Silica can not only reduce the friction between rubber molecules but also fill the gap between the rubber molecular chains, thus affording low roll resistance, high wear resistance, and low energy consumption for rubber materials [15,16,17,18,19]. However, the silica has a small particle size, and its surface contains abundant hydroxyl groups, resulting in the easy adsorption and aggregation of common tread rubbers, such as natural rubber (NR), styrene butadiene rubber (SBR), and butadiene rubber (BR) [20,21,22]. The addition of certain functional groups is of crucial significance to improve the property characteristics of non-polar polymers or enhance their compatibility with some polar fillers [23].

Epoxidized natural rubber (ENR) has attracted increasingly attention in high-performance tire tread rubber due to its excellent airtightness, good oil resistance, and higher adhesion to metals or rubbers than the NR [24,25,26]. Mohd and his coworkers explored the effects of vulcanization temperature on the sulfurization properties and vulcanization kinetics of ENR compounds, which revealed that the carbon black-filled ENR had higher activation energies than carbon black-filled NR compounds [27]. The silica-filled ENR compounds were evaluated for truck tire tread, which showed high chemically bound interaction and high Akron abrasion index. And the use of silica-filled ENR compounds in tread compound was acknowledged as a renewable material for preparing green tire applications [28]. Additionally, ENR was also used as a compatibilizer for silica and non-polar rubbers such as NR [29] and SBR [30] due to the existence of epoxy groups in ENR chains. Although the silica-reinforced ENR compounds rubbers are characterized with high wet slip resistance, low heat generation, and low rolling resistance, their mechanical strength is significantly lower than the carbon black-reinforced ENR. Omar et al. studied the effect of silica/carbon black hybrid fillers on ENR compounds with Si69 as a coupling agent [31]. With the addition of carbon black, the aggregation of silica particles in the ENR matrix was reduced, and the resulting ENR vulcanizates showed improved mechanical properties and winter traction performance. However, these reported studies mainly investigated the macroscopic mechanical properties of ENR compounds rather than the microscopic structural changes and interfacial interaction, and the characterization of the microstructures is not comprehensive. Meanwhile, the use of coupling agents easily generates volatile organic compounds (VOC) at volcanizing temperatures, which is harmful to human health.

In this work, we explored a CB/silica hybrid reinforcing strategy to prepare high-performance ENR-based green tread rubbers. In the absence of any coupling agent, we greatly enhanced the interchain force between the hybrid fillers and ENR molecular chains by adjusting the blending ratios of CB/silica and improved the dispersion uniformity of the hybrid fillers in the ENR matrix. We made a detailed analysis about the effects of the CB/silica ratios on the vulcanization characteristics, physical mechanical properties, dynamic mechanical properties, cross-linking density, and damping performance. The experimental results indicated that when the total amount of CB and silica was constant, the dispersion uniformity of silica in the ENR matrix was evidently enhanced with increasing addition of silica, and both the sulfurization performance and physical mechanical properties were improved. Moreover, as the vulcanization temperature increased, the grafting efficiency of silica on the ENR molecular chains was enhanced, which satisfied the requirements of high-performance green tires with low rolling resistance, high wear resistance, high strength, low heat generation, high heat resistance, and good wet skid resistance.

## 2. Experimental Section

### 2.1. Materials

Epoxidized natural rubber (ENR) with 20% epoxy groups was provided by the Chinese Academy of Tropical Agricultural Sciences. The ENR has a density of 0.97 g/cm^3^ and a Mooney viscosity of 70–90 at ML (1 + 4) 100 °C. Silica (1165 MP) with a diameter of 250 μm and specific surface area (BET) of 115 m^2^/g was purchased from Solverodia Silica (Qingdao, China) Co., Ltd. CB (N660) was purchased from Cabot Corporation, USA. The di-n-butyl phthalate (DBP) of the CB was 90 ± 6 cm^3^/100 g, and the adsorption-specific surface area under nitrogen was 29–41 m^2^/g. Other processing aids (such as zinc oxide, stearic acid, antioxidant 4020, etc.) and solvents (such as dichloromethane) were commercially purchased and used without further purification.

### 2.2. Preparation of ENR Compounds with Different Silica/CB Ratios

First, the ENR was plasticized on a two-roll mill with a rotor speed of 60 rad/min at 80 °C. After that, the ENR was mixed in an internal mixer for 1 min, followed by the sequential addition of steric acid (SA), zinc oxide (ZnO), antioxidant 4020, and accelerator RD and continuous stirring for 2 min. Then, the silica, CB, thermoplastic adhesive resin (SP1068), and aromatic oil (V700) were introduced in this internal mixer and continuously stirred for 5 min. Upon reaching 105 °C, the mixed rubber compounds were taken out from the internal mixer and cooled to room temperature. The insoluble sulfur OT20, accelerator NS, accelerator DTDM, and accelerator DPG were added into the rubber compounds on the two-roll open mixer, followed by being cut three times and rolled five times. The processing formula of ENR compounds in this study is shown in Table 1.

### 2.3. Preparation of ENR Vulcanizates with Different Silica/CB Ratios

The mixed ENR compounds were placed at room temperature for more than 12 h. After that, the ENR vulcanizates were prepared in a plate curing press machine at 145 °C for a time to the optimum cure time (tc90) obtained from the curing curve detected on a GT-M2000-A (Alpha Technologies, Hudson, NY, USA) vulcameter under a pressure of approximately 15 MPa.

### 2.4. Vulcanization Characteristic Analysis

In the vulcanization process, the torque is directly proportional to the cross-linking density. The vulcanization rate can be characterized by the change rate of the torque according to the following equation [32,33]:

(1) $$V=\frac{dln\left(M_{H}-M_{t}\right)}{dt}={K(M_{H}-M_{t})}^{n}$$

where $M_{H}$ represents the maximum torque, $M_{t}$ represents the curing time at the time of *t*, *K* represents a rate constant, and *n* represents the reaction order.

For the first-order reaction, namely, *n* = 1, the integration of Equation (1) is as follows:

(2) $$\ln\left(M_{H}-M_{t}\right)=lnA-K_{t}$$

According to the reaction model of scorch period derived from Coran, the curing rate can be determined from the following equation:

(3) $$-(\alpha K_{3}/K_{4})ln[(K_{2}e^{K1t}-K_{1}e^{K2t})/(K_{2}-K_{1})]$$

(4) $$v_{ut}=V_{u\propto }[1-e^{-K2(t-ti)}]$$

Additionally, the reaction during the burning period is not a first-order reaction. The true first-order reaction begins at $t_{dis}$, when the change rate in the torque reaches its maximum value. Therefore, the vulcanization kinetics after $t_{dis}$ are expressed as:

(5) $$Ln\left(M_{H}-M_{t}\right)=lnA-K(t-t_{dis})$$

According to the Arrhenius formula, the relationship between reaction rate constant and vulcanization temperature can be expressed as:

(6) $$K=Ae^{-Ea/RT}$$

where *K* is the reaction rate constant, *R* is the gas constant (8.1345 J mol^–1^ K^–1^), *T* is the absolute temperature, and *Ea* is the activation energy of reaction (kJ/mol).

By taking the natural logarithm at both ends of Equation (6), we can obtain the following equation:

(7) $$LnK=\left(-\frac{Ea}{RT}\right)+lnA$$

The activation energy of the reaction can be determined from the relationship between *LnK* and the reciprocal of temperature (1/*T*).

### 2.5. Measurements

Physical mechanical properties: The tensile strength of ENR vulcanizes was measured on a GT-AT-7000M (Goodtechwill, Taiwan, China) material testing instrument at a speed of 500 mm/min. ENR vulcanizes were cut into a dumbbell shape with a thickness of 2 mm according to ISO 37-2024 [34]. The tear strength of ENR vulcanizates was tested on a GT-AT-7000M (Goodtechwill, Taiwan, China) material testing instrument at a speed of 500 mm/min. ENR vulcanizates were cut into a right-angle shape with a thickness of 2 mm according to ISO 34-1:2022 [35]. The hardness of ENR vulcanizates was tested on a shore A durometer (Frank Bacon Machinery Sales Company, Warren, MI, America) according to ISO 18517:2015 [36]. DIN abrasion was detected on a GT-7012-D (Goodtechwill, Qingdao, China) abrasion tester according to ISO 4649:2024 [37]. The resilience was characterized on a GT-7042-RE (Goodtechwill, Qingdao, China) elastic testing machine according to ISO 4662:2017 [38]. The samples were made into a flat cylinder shape. For the above measurements, at least five specimens of each sample were measured for obtaining an average value.

Compression fatigue test: The compression fatigue was detected on a GT-RH-2000N (Goodtechwill, Qingdao, China) rubber compression heat generating test machine according to ISO 4666-3-2022 [39]. The test was conducted at a temperature of 55 ± 1 °C, a stroke of 5.71 ± 0.03 mm, and a load of 1.00 ± 0.03 MPa.

Differential scanning calorimetry (DSC): The glass transition temperature (Tg) was obtained on a TA DSC-Q20 thermal analyzer (TA Instruments, Shanghai, China) over a temperature range from –80 to 200 °C at a heating rate of 10 °C/min under a nitrogen atmosphere.

Dynamic mechanical analysis (DMA): The dynamic mechanical properties of ENR vulcanizates were analyzed using a DMAQ800 Dynamic Viscoelastic Spectrometer (TA Instruments, Shanghai, China) over a temperature range from –80 to 80 °C at a heating rate of 3 °C/min and a scanning frequency of 10 Hz. The length, width, and height of the test sample were 10 mm, 4 mm, and 2 mm, respectively.

Rubber processing analysis (RPA): The dynamic viscoelasticity of ENR compounds was obtained on a rubber processing analyzer (Alpha Technologies Inc., Hudson, OH, USA) over a strain range of 1–100% at a temperature of 60 °C and a frequency of 60 Hz.

Scanning electron microscopy (SEM): The fracture surfaces of the tensile samples were studied using a JSM-6700F microscope (Japanese Electronics Co. Ltd., Beijing, China) at a filament voltage of 20 KV. Before testing, the fracture surfaces of the samples required be sputter-coated with a thin layer of gold.

Fourier transform infrared (FTIR) spectra: The elemental compositions of ENR were analyzed using a Nicolet FTIR-Magna-750 spectrophotometer (Thermo Nicolet Corporation, Massachusetts, Waltham, MA, USA) in the range of 4000~400 cm^–1^ by total reflection mode.

Low-field solid-state nuclear magnetic: The cross-linking density of ENR vulcanizates was detected using a nuclear magnetic cross-linked density analyzer with a magnetic field intensity of 0.35 T and a resonant frequency of 15.135 MHz.

## 3. Results and Discussion

### 3.1. Vulcanization Characteristics of ENR Compounds

**Figure 1:**
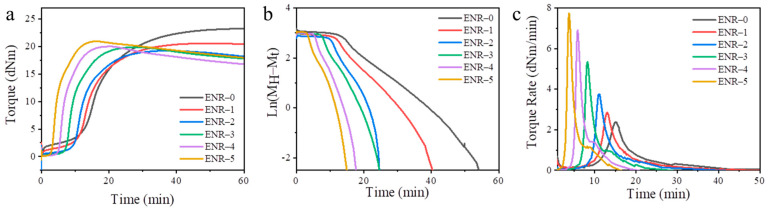
(**a**) Torque, (**b**) ln(*M_H_* − *M_t_*), and (**c**) torque rate versus vulcanization time for ENR with different CB/silica ratios.

According to the preparation formula of ENR vulcanizates, the vulcanization temperature was set at 143 °C. The prepared rubber compounds were vulcanized on MD-R2000 to obtain the curing curve in Figure 1.

As the addition of silica decreased, the scorch time of ENR was prolonged, the vulcanization rate was slowed down, and the slope of the curing curve was reduced, indicating a delayed vulcanization starting point and a slow vulcanization rate (Figure 1a). This was because, compared to the CB, the silica had a higher acidic nature caused by the siloxane and silanol groups on its surface. Additionally, the maximum torque of ENR compounds gradually increased with increasing content of silica, which was because the reaction between the hydroxyl groups on the silica surface and the epoxy group on the ENR resulted in an increase in the cross-linking density.

According to Equation (2), we plotted ln(*M_H_* − *M_t_*) versus curing time (t) to explore the effects of silica content on the vulcanization rate of ENR. As seen from Figure 1b, the vulcanization process of the ENR was composed of a scorch period and a hot vulcanization period. The hot vulcanization period was divided into two stages. The first stage showed obvious first-order reaction characteristics, and the reaction rate constant (K) could be calculated from the slope or linear fitting. The second stage was relatively complex and did not conform to the characteristics of a first-order reaction. In the following description, we referred to the two stages as the first and second stages of vulcanization.

As mentioned above, the determination of t_dis_ is the key to accurately solving the rate constant K. The t_dis_ refers to the vulcanization time, which corresponds to the maximum vulcanization rate (V_m_). Thus, the V_m_ versus curing time can reveal the effects of the silica content on the t_dis_. As shown in Figure 1c, the vulcanization process was divided into two stages: the increase and decrease in vulcanization rate. As the addition of silica increased, the V_m_ of ENR gradually decreased, and the t_dis_ was also prolonged. The t_dis_ and V_m_ of ENR at different temperatures were summarized in Table 2. As the vulcanization temperature increased, the t_dis_ gradually decreased, and the V_m_ gradually increased, which indicated that the high temperature was beneficial for enhancing the vulcanization speed.

In the vulcanization process of ENR compounds, the first stage of the vulcanization was composed of two first-order reactions (n = 1). According to Equation (2), we explored the ln(*M_H_* − *M_t_*) versus vulcanization time for the first stage (starting from t_dis_), which could fit the rate constant K of the first vulcanization stage. The detailed data are summarized in Table 3.

**Figure 2:**
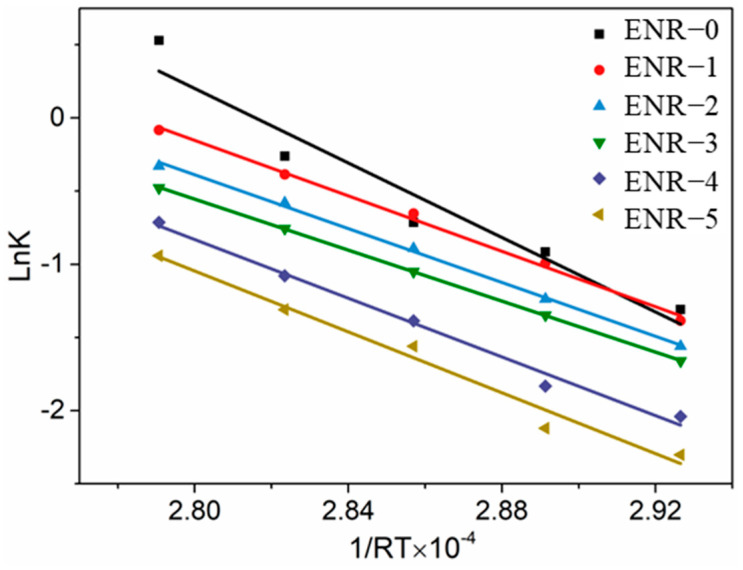
Reaction rate constant versus vulcanization temperature for ENR compounds at the first vulcanization stage.

According to the reaction rate constant K from Table 3, we explored the reaction rate constant versus the reciprocal of vulcanization temperature based on the Arrhenius Equation (7). The corresponding data curves are plotted in Figure 2.

As seen from Figure 2, the reaction rate constant versus vulcanization temperature of the ENR prepared with different CB/silica ratios exhibited a good linear fitting relationship. The activation energies of the ENR are summarized in Table 4.

It can be seen from Table 4 that for the first vulcanization stage, the vulcanization activation energy decreased first and then increased with the increasing addition of silica. This illustrated that the addition of silica accelerated the vulcanization speed of the ENR compounds. But when excessive silica (such as CB/silica = 10:40) was added into the ENR compounds, the vulcanization speed began to decrease.

**Figure 3:**
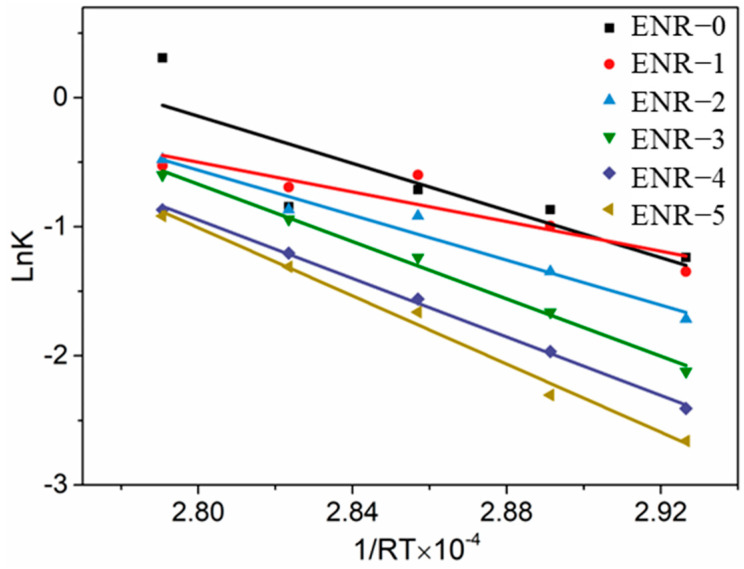
Reaction rate constant versus vulcanization temperature for ENR at the second vulcanization stage.

Similarly, we explored the ln(*M_H_* − *M_t_*) versus vulcanization time for the second vulcanization stage. The vulcanization rate constant of the ENR at different vulcanization temperatures is summarized in Table 5. We also investigated the reaction rate constant versus the reciprocal of vulcanization temperature based on the Arrhenius equation (Figure 3) and determined the activation energy of the ENR at the second vulcanization stage in Table 6.

As shown in Table 6, the activation energy of ENR at the second vulcanization stage decreased first and then increased with increasing content of silica. When the addition of silica exceeded 30 phr, namely, CB/silica = 20:30, the activation energy began to increase (which was higher than the ENR with only CB), and thus, the vulcanization speed was obviously reduced.

### 3.2. Reaction Mechanism Analysis

**Figure 4:**
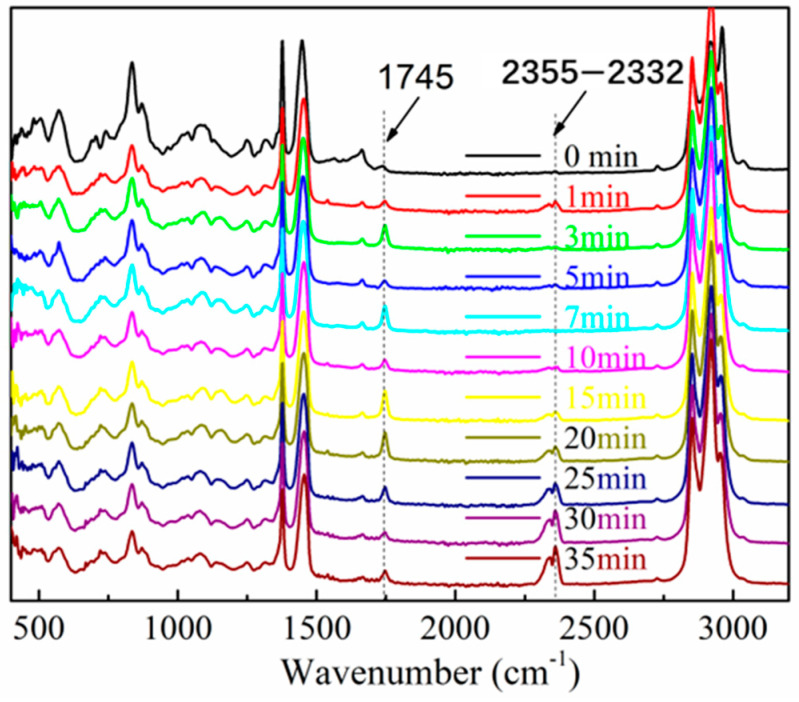
FTIR spectra of ENR with only addition of silica (50 phr) under different vulcanization times.

**Figure 5:**
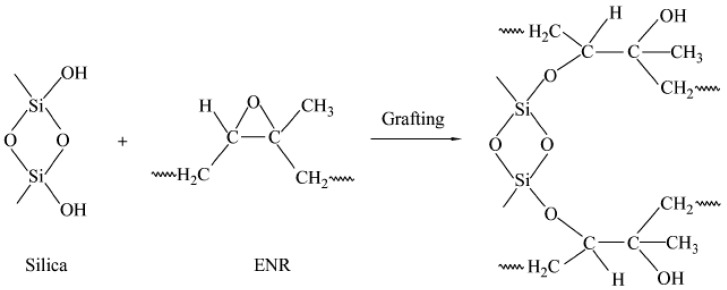
Grafting reaction of silica with ENR.

The ENR compounds with only the addition of silica were vulcanized at 143 °C under different vulcanization times. After that, the obtained ENR vulcanizates were immersed in the dichloromethane solvents for 12 h and dried in a bake oven at 100 °C for 2 h. The purified ENR vulcanizates were used for the FTIR analysis. As shown in Figure 4, the characteristic bands at 1100 cm^–1^ and 800 cm^–1^ were assigned to the Si–O–Si asymmetric stretching vibration and Si–O symmetric stretching vibration, respectively. After the reaction of ENR and silica, there appeared C–H, C=C, C=O, and Si–O absorption peaks in the FTIR spectra of ENR compounds, and no obvious absorption peak at 870 cm^–1^ attributing to the epoxy groups of ENR was observed. These results indicated that the epoxy groups on the ENR chains could react with the silicon hydroxyl groups on the silica surface (Figure 5) and form a strong interaction force (hydrogen bond or chemical bond), which was beneficial for enhancing the dispersion of silica in the ENR matrix.

### 3.3. Physical Mechanical Properties

The physical and mechanical properties of ENRs with different CB/silica ratios are shown in Table 7. The ENR without the addition of reinforcing fillers, namely, ENR-0, had a low tensile strength of only 8.5 MPa. By contrast, the ENR with different CB/silica ratios showed a multiplied increase in tensile strength, tensile stress, hardness, and density. As the content of silica increased, the tear strength and density of the ENR were significantly enhanced, which was because the improved compatibility between ENR chains and silica reduced the filler/polymer chain distances and enhanced the dispersion of fillers in the polymer matrix. Moreover, as the contents of silica increased, the ENR exhibited significantly reduced heat generation and improved wear resistance, which satisfied the requirements of high-performance green tires.

After aging for 48 h at 100 °C, all samples’ tensile stress and elongation at break decreased, while the shore A hardness increased. This was because the free radicals generated during thermal aging caused excessive cross-linking of ENR molecular chains. The decrease in the movement ability of the ENR chain segments results in a reduction of dissipated energy, thus degrading the tensile strength and elongation at break of ENR compounds. However, due to the short chain segments and reduced movement ability, the hardness and tensile stress were improved. Additionally, the tensile retention rate of ENR-3 reached the highest among the test samples, indicating that the aging resistance of ENR compounds is the best when the CB/Silica was 30:20. This was because the CB particles could be uniformly dispersed in the ENR matrix with the assistance of silica, which increased the bond rubber content and achieved an outstanding aging resistance.

### 3.4. Nuclear Magnetic Resonance (NMR) Cross-Linking Density

The addition of silica resulted in the change in the microstructures and molecular movements in the ENR vulcanizates. The linear structures of the ENR transformed into the cross-linked structures upon addition of silica, and the molecular chains were cross-linked with each other, thereby resulting in a change in the molecular weight, cross-linking density, and vulcanization temperature. The NMR crosslinking density of ENR vulcanizates with different CB/silica ratios is shown in Table 8. As the addition of silica increased, the total cross-linking density of ENR compounds was reduced, and the average molecular weight of the cross-linked molecular chains (Mc) was enhanced. The -OH groups on silica could absorb some curing agents. Although ENR-5 had a lower cross-linking density, its tensile strength and elongation at break were the highest among these test samples, which was attributed to the strong interfacial interaction between ENR and silica. The transverse relaxation time (T_2_) was dependent on the molecular movement ability above the glass transition temperature, and the relaxation time was cross-linking chain < dangling chain. Compared with the ENR compounds without CB/silica hybrid fillers, the ENR compounds with CB/silica hybrid fillers displayed obviously higher T_2_, indicating that the addition of CB/silica hybrids could increase the amounts of dangling chains.

### 3.5. Glass Transition Temperature (Tg) of ENR

**Figure 6:**
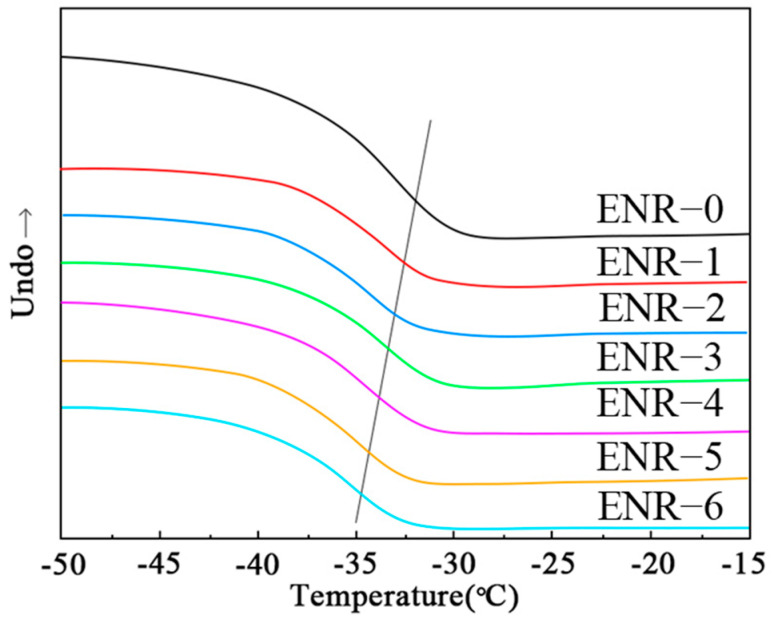
DSC curves of ENR with different CB/silica ratios.

The effects of silica molecules on the Tg of ENR remain controversial. Therefore, we further explored the effect of different CB/silica ratios on the Tg of ENR. The test results are summarized in Figure 6. The Tg is a reflection of the movement capacity of the chain segment, which is irrelevant to cross-link density. As the contents of silica increased, the Tg of the ENR shifted toward a low-temperature region, which was because the addition of silica increased the ENR interchain distance, thereby enhancing the movement ability of molecular chains. Additionally, the ring opening of the epoxy groups could also increase the flexibility of chain segments. A more flexible chain segment enables it to dissipate more energy during the force-bearing process, thereby enhancing its mechanical properties.

### 3.6. Dynamic Mechanical Analysis (DMA) of ENR

**Figure 7:**
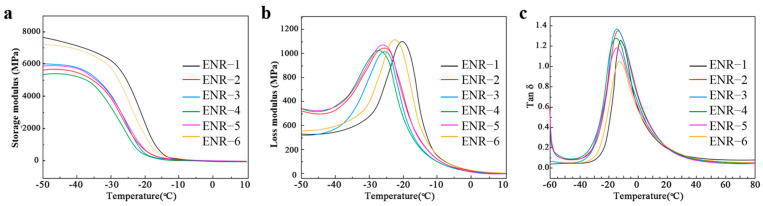
(**a**) Storage modulus, (**b**) loss modulus, and (**c**) loss factor versus temperature of ENR with different CB/silica ratios.

Compared to ENR with only the addition of CB or silica, the ENR with CB/silica hybrid fillers showed slightly reduced elastic modulus and storage modulus (Figure 7a), which was because the blending of CB and silica affected the construction of the filler network in the bound rubber, and the grafting of silica on ENR chains resulted in an evident decrease in its elastic modulus. Similarly, the ENR with CB/silica hybrid fillers showed a degraded irritability at low temperature and an improved irritability at high temperature (Figure 7b), which indicated that the ENR with CB/silica hybrid fillers had excellent loss modulus and thus enhanced the responsiveness to energy.

It can be seen from Figure 7c and Table 9 that as the contents of silica decreased, the ENR vulcanizates showed the first increase and then decrease in tan δ value. Thereinto, when the addition of silica was 10 phr (ENR-2), the tan δ value of the ENR vulcanizates reached a maximum and the effective damping temperature range (tan δ > 0.3) was broadened. Meanwhile, the ENR vulcanizates with 20 phr (ENR-3) of silica displayed a high tan δ value at 0–30 °C and the lowest tan δ value at 60–80 °C, which indicated that the addition of silica could improve the wet and dry slip resistance as well as reduce the rolling resistance and heat generation of ENR vulcanizates.

### 3.7. Rubber Processing Analysis (RPA) of ENR

CB and silica particles aggregated in the ENR matrix to form a cross-linked network structure, which generated hysteresis heating at low shear strains, leading to an increase in automobile exhaust emissions [40]. The particle size of silica was smaller than that of CB. But the silica could be uniformly dispersed in the ENR matrix. As the vulcanization temperature increased, many filler networks were further aggregated. This phenomenon was called flocculation. We adopted the reaction of silica with the epoxy groups on ENR chains to enhance the dispersion uniformity of silica into the ENR matrix. The polymer-silica interfacial characteristics affected the movement of the fillers in the ENR vulcanizates when the rubber materials were subjected to deformations.

**Figure 8:**
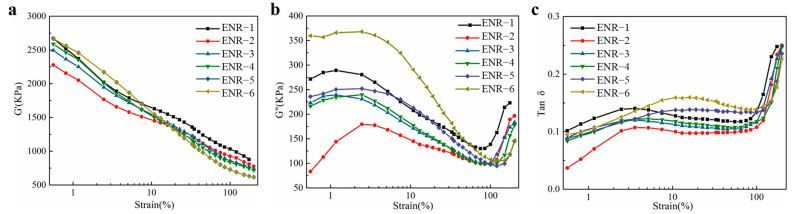
(**a**) Storage modulus (G′), (**b**) loss modulus (G″), and (**c**) tan δ versus shear strains for ENR with different CB/silica ratios.

As the addition of silica increased, the amount of silica molecules grafted on ENR chains increased, which improved the interaction between silica and ENR molecules, thus affording ENR with a higher energy storage modulus and loss modulus. As shown in Figure 8, compared to the ENR with CB/silica hybrid fillers, the ENR with only CB exhibited higher storage modulus and loss modulus, which was because it was easy to form a core–shell structure between CB and ENR molecular chains, thereby increasing the content of bound rubber and improving the modulus of ENR compounds. However, the particle size of silica was smaller than that of CB, and the silica particles could be uniformly dispersed into the ENR matrix, which thus increased the loss factor (tan δ = G″/G′).

### 3.8. Scanning Electron Microscopy (SEM) of ENR

**Figure 9:**
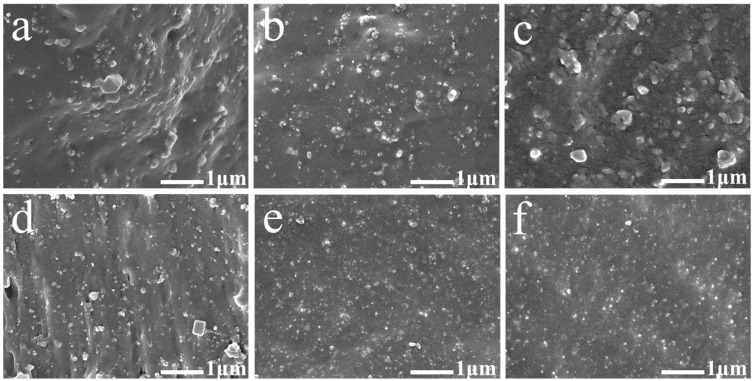
SEM images of ENR with different CB/silica ratios. (**a**) ENR-1, (**b**) ENR-2, (**c**) ENR-3, (**d**) ENR-4, (**e**) ENR-5, and (**f**) ENR-6.

We employed the SEM images to analyze the dispersion of CB/silica hybrid fillers into the ENR matrix, as shown in Figure 9. For ENR with a high CB/silica ratio (50:0~30:20), it was obvious that the CB particles were aggregated in the ENR matrix and the large-size CB particles were layered with the rubber interface. With the decrease in the CB/silica ratios, namely, the increase in silica, the obvious aggregation of CB particles gradually disappeared. Specifically, when the CB/silica ratio was 10:40, no uneven dispersion was observed, which was because the silica had a strong interfacial interaction and compatibility with the ENR chains, thus enhancing the dispersion uniformity of CB/silica hybrid fillers into the ENR matrix.

## 4. Conclusions

In summary, we developed an effective CB/silica hybrid reinforcing system by optimizing its blending ratio and explored the effects of CB/silica ratios on the vulcanization characteristics, physical mechanical properties, and dynamic mechanical performances of ENR compounds. As revealed by the FTIR analysis, the silica molecules could be grafted on the ENR molecular chains, which significantly enhanced the dispersion uniformity of hybrid fillers into the ENR matrix. Meanwhile, the introduction of silica can also enhance the dispersity and reinforcing effect of CB particles on the ENR compounds. Compared to the ENR with only CB fillers, those with CB/silica hybrid fillers showed simultaneously improved mechanical strength, tensile strain, wear resistance, resilience, oxidation resistance, and damping properties, as well as reduced heat generation and rolling resistance. For example, compared to the ENR vulcanizates with only CB fillers, the tensile strain of the ENR with CB/silica (30:20) hybrids increased by 20%, and the tensile retention rate after aging for 48 h at 100 °C increased by 20%. Our research results indicated that by tuning the CB/silica blending ratio, the prepared ENR vulcanizates can satisfy the requirements of high-performance green tread rubber materials.

## Figures and Tables

**Table 1:** Processing formula for ENR compounds in this work.

Materials	ENR-0	ENR-1	ENR-2	ENR-3	ENR-4	ENR-5	ENR-6
ENR	100	100	100	100	100	100	100
CB	0	50	40	30	20	10	0
Silica	0	0	10	20	30	40	50
ZnO	7.5	7.5	7.5	7.5	7.5	7.5	7.5
SA	1	1	1	1	1	1	1
4020	3	3	3	3	3	3	3
RD	0.75	0.75	0.75	0.75	0.75	0.75	0.75
SP1068	2	2	2	2	2	2	2
V700	2	2	2	2	2	2	2
OT20	3.18	3.18	3.18	3.18	3.18	3.18	3.18
NS	1.2	1.2	1.2	1.2	1.2	1.2	1.2
DTDM	0.8	0.8	0.8	0.8	0.8	0.8	0.8
DPG	0.2	0.2	0.2	0.2	0.2	0.2	0.2
T0TAL	171.6	171.6	171.6	171.6	171.6	171.6	171.6

**Table 2:** t_dis_ and V_m_ values of ENR with different CB/silica ratios under different temperatures.

Vulcanization Index	Sampe	138 °C	143 °C	148 °C	153 °C	158 °C
t_dis_ (min)	ENR-1	5.43	3.83	2.84	2.06	1.58
	ENR-2	8.30	5.91	4.36	3.20	2.39
	ENR-3	11.52	8.30	5.93	4.27	3.13
	ENR-4	15.23	11.14	7.39	5.34	3.79
	ENR-5	17.22	13.04	8.64	6.11	4.16
	ENR-6	19.22	15.13	9.14	6.67	4.57
V_m_ (dNm/min)	ENR-1	5.71	7.14	9.52	12.41	15.71
	ENR-2	5.19	6.92	9.04	11.54	14.67
	ENR-3	4.00	5.33	6.78	9.09	12.03
	ENR-4	2.94	3.75	5.70	7.36	9.69
	ENR-5	2.31	2.83	3.99	5.98	7.22
	ENR-6	1.82	2.36	4.01	4.76	7.44

**Table 3:** Reaction rate constants of ENR with different CB/silica ratios at the first vulcanization stage.

Sample	138 °C	143 °C	148 °C	153 °C	158 °C
ENR-1	0.27	0.40	0.49	0.77	1.7
ENR-2	0.25	0.37	0.52	0.68	0.92
ENR-3	0.21	0.29	0.41	0.56	0.72
ENR-4	0.19	0.26	0.35	0.47	0.62
ENR-5	0.13	0.16	0.25	0.34	0.49
ENR-6	0.10	0.12	0.21	0.27	0.39

**Table 4:** Activation energy of ENR with different CB/silica ratios at the second vulcanization stage.

Sample	ENR-1	ENR-2	ENR-3	ENR-4	ENR-5	ENR-6
Ea (kJ/mol)	12.7	9.5	9.2	8.7	10.3	10.4

**Table 5:** Reaction rate constants of the second vulcanization stage.

Sample	138 °C	143 °C	148 °C	153 °C	158 °C
ENR-1	0.29	0.42	0.49	0.43	1.36
ENR-2	0.26	0.37	0.55	0.50	0.59
ENR-3	0.18	0.26	0.40	0.42	0.62
ENR-4	0.12	0.19	0.29	0.39	0.55
ENR-5	0.09	0.14	0.21	0.30	0.42
ENR-6	0.07	0.10	0.19	0.27	0.40

**Table 6:** Activation energy at the second vulcanization stage for ENR with different CB/silica ratios.

Sample	ENR-1	ENR-2	ENR-3	ENR-4	ENR-5	ENR-6
Ea (kJ/mol)	9.1	8.7	5.7	11.1	11.3	13.2

**Table 7:** Physical mechanical properties of ENR with different CB/silica ratios.

Property Index	ENR-0	ENR-1	ENR-2	ENR-3	ENR-4	ENR-5	ENR-6
Tensile strength (MPa)	8.5	22.8	24.5	25.1	26.4	27.0	28.8
Elongation at break (%)	440	436	476	523	547	556	567
Tear strength (kN·m^–1^)	34.4	45.6	49.0	52.6	51.1	52.5	56.9
Tensile stress at 100% (MPa)	1.2	3.5	3.3	2.8	2.5	2.6	2.6
Tensile stress at 300% (MPa)	3.4	15.1	15.1	12.6	12.4	12.8	11.9
Shore A hardness (°)	48	68	68	67	67	66	67
Density (g·cm^–3^)	1.021	1.181	1.187	1.191	1.195	1.200	1.206
Resilience (%)	34	43	40.5	40.5	39	41	41
Compression fatigue (temperature rise) (°C)	11.5	14.3	12.3	12.6	12.25	13.1	12.8
DIN abrasion (cm^3^)	172	201	174	199	193	188	168
Aging for 48 h at 100 °C							
Tensile strength (MPa)	1.8	9.7	8.3	9.8	9.0	8.8	9.3
Elongation at break (%)	89	94	84	96	87	94	108
Tensile stress at 100% (MPa)	1.6	8.8	8.5	8.1	8.1	7.8	7.2
Shore A hardness (°)	52	78	78	76	75	75	79
Tensile retention rate (%)	20.8	32.7	33.8	39.0	33.9	29.0	32.2
Elongation at break (%)	17.2	19.9	17.6	18.4	16.0	16.8	19.1

**Table 8:** NMR cross-linking density of ENR vulcanizates with different CB/silica ratios.

	ENR-0	ENR-1	ENR-2	ENR-3	ENR-4	ENR-5
Total cross-linking density (kmol/cm^3^)	21.2	20.2	18.7	17.6	16.2	15.1
Mc (kg/mol)	5.56	6.1	6.4	6.8	7.4	8.0
T_2_ (ms)	1.9	2.0	1.8	2.2	2.2	2.1
qM_2_ (×10^4^/s^2^)	149.9	128.8	114.2	99.9	84.7	72.0

**Table 9:** DMA characteristics of ENR with different CB/silica ratios.

Sample	tan δ (0 °C)	tan δ (30 °C)	tan δ (60 °C)	tan δ (80 °C)	tan δ_max_	Temperature at tan δ_max_ (°C)
ENR-1	0.598	0.191	0.085	0.079	1.26	–12.1
ENR-2	0.739	0.203	0.064	0.051	1.35	–13.7
ENR-3	0.701	0.195	0.054	0.042	1.38	–14.6
ENR-4	0.646	0.194	0.067	0.055	1.28	–15.3
ENR-5	0.618	0.183	0.068	0.058	1.19	–14.6
ENR-6	0.615	0.195	0.078	0.066	1.05	–12.9

## Data Availability

The original contributions presented in the study are included in the article, further inquiries can be directed to the corresponding author.
